# Wastewater-based monitoring of SARS-CoV-2 at UK airports and its potential role in international public health surveillance

**DOI:** 10.1371/journal.pgph.0001346

**Published:** 2023-01-19

**Authors:** Kata Farkas, Rachel Williams, Natasha Alex-Sanders, Jasmine M. S. Grimsley, Igor Pântea, Matthew J. Wade, Nick Woodhall, Davey L. Jones

**Affiliations:** 1 Centre for Environmental Biotechnology, School of Natural Sciences, Bangor University, Bangor, Gwynedd, United Kingdom; 2 School of Ocean Sciences, Bangor University, Menai Bridge, Anglesey, United Kingdom; 3 Data, Analytics, and Surveillance Group, UK Health Security Agency, London, United Kingdom; 4 The London Data Company, London, United Kingdom; 5 School of Engineering, Newcastle University, Newcastle-upon-Tyne, United Kingdom; 6 Food Futures Institute, Murdoch University, Murdoch, Australia; Fundacao Oswaldo Cruz, BRAZIL

## Abstract

It is well established that air travel plays a key role in the global spread of many enteric and respiratory diseases, including COVID-19. Even with travel restrictions (e.g. mask wearing, negative COVID-19 test prior to departure), SARS-CoV-2 may be transmitted by asymptomatic or pre-symptomatic individuals carrying the virus. Due to the limitation of current clinical surveillance approaches, complementary methods need to be developed to allow estimation of the frequency of SARS-CoV-2 entry across international borders. Wastewater-based epidemiology (WBE) represents one such approach, allowing the unbiased sampling of SARS-CoV-2 carriage by passenger cohorts entering via airports. In this study, we monitored sewage in samples from terminals (n = 150) and aircraft (n = 32) at three major international airports in the UK for 1–3 weeks in March 2022. As the raw samples were more turbid than typical municipal wastewater, we used beef extract treatment followed by polyethylene glycol (PEG) precipitation to concentrate viruses, followed by reverse transcription quantitative PCR (RT-qPCR) for the detection of SARS-CoV-2 and a faecal indicator virus, crAssphage. All samples taken from sewers at the arrival terminals of Heathrow and Bristol airports, and 85% of samples taken from sites at Edinburgh airport, were positive for SARS-CoV-2. This suggests a high COVID-19 prevalence among passengers and/or airport staff members. Samples derived from aircraft also showed 93% SARS-CoV-2 positivity. No difference in viral prevalence was found before and after COVID-19 travel restrictions were lifted. Our results suggest that WBE is a useful tool for monitoring the global transfer rate of human pathogens and other disease-causing agents across international borders and should form part of wider international efforts to monitor and contain the spread of future disease outbreaks.

## 1. Introduction

Since the start of the COVID-19 pandemic in March 2020, SARS-CoV-2 has been responsible for over 0.5 billion confirmed cases and over six million deaths worldwide [1]. Approximately 40–45% are asymptomatic or associated with mild symptoms, hence, the number of cases based on clinical surveillance is typically underestimated [2–4]. Although SARS-CoV-2 is a respiratory pathogen, ca. 43% of symptomatic patients shed the virus in their faeces [5] and the virus has also been detected in the faeces of asymptomatic and mildly symptomatic infected individuals [6, 7]. Due to the high prevalence of the virus in faeces, the RNA genome of SARS-CoV-2 can be detected and quantified in domestic wastewater. Hence, by using wastewater-based epidemiology (WBE), the temporal dynamics of viral RNA concentrations in wastewater can be measured and, subsequently, related to case numbers [8]. It has also been shown that WBE can be used as an early warning system and as a predictive approach for the monitoring and mitigation of the COVID-19 pandemic [8–11]. As WBE can provide unbiased information at the community level, it has been implemented in many countries as a complementary monitoring tool for COVID-19 and other viral diseases [12–15]. Furthermore, WBE has been successfully utilised to support monitoring of highly transient populations in near-source settings at the building scale, such as university campuses [16–18], suggestive of its utility to monitor smaller, localised, and dynamic populations.

The usefulness of WBE for international border control has also been investigated, focusing on airplane wastewater surveillance, suggesting that SARS-CoV-2 can be detected, quantified, and sequenced in a static setting distinct from classical piped flows (i.e., sewer networks) [19–21]. There has been only a single study, to date, reporting SARS-CoV-2 RNA levels in sewage from a wastewater treatment plant associated with a major airport [9]. However, there is still no comprehensive, spatio-temporal study of the value of WBE for airport-associated public health assessment.

As data quality predominantly depends on the efficiency of sample processing, several studies have investigated the usefulness of different wastewater concentration methods for SARS-CoV-2 detection [22–27]. To date, filtration, ultrafiltration, and precipitation methods have been widely used for COVID-19 WBE [28–31]. However, the viral recoveries also depend on the volume of the samples and the physico-chemical properties of the wastewater [32, 33]. As the composition of wastewater near-source and at specific locations with water-preserving waste systems (e.g., aircrafts, ferries, mobile toilets) is often more concentrated than the sewage collected at wastewater treatment plants, the currently used WBE methods may need to be modified to optimise performance.

The aim of this study was to investigate the value of WBE for COVID-19 monitoring and public health protection, at three airports in the United Kingdom. Samples were collected from sewers close to the airport terminals, from vacuum trucks collecting wastewater from incoming aircraft, and at a wastewater treatment plant (WWTP) in the vicinity of the airport. We also trialled different wastewater concentration methods tailored for highly turbid samples with the best method coupled with RT-qPCR for viral RNA quantification.

## 2. Methods and materials

### 2.1 Sample collection and spiking

Wastewater sampling was conducted by external partners (2030 Labs, Veolia and Aqua Enviro, UK) in liaison with airport staff. Sampling was authorised by Heathrow Airport Ltd, Bristol Airport Ltd and Edinburgh Airport Ltd.

#### 2.1.1 Set 1—Spiked samples for method validation

For method validation, 24 1-litre grab wastewater samples were collected at Edinburgh airport on the 7^th^ and 8^th^ March 2022. From the 24 samples, six 4-litre composites were created by mixing. Four of the composite mixtures were spiked with different concentrations of heat-inactivated SARS-CoV-2 Wuhan and Alpha variants (provided by Prof Andrew Weightman, Cardiff University) to reach final concentrations of approximately 104–10^5^ genome copies (gc)/ml (Table 1). After thorough mixing, approximately 220 ml aliquots were prepared and processed in triplicates as described below.

**Table 1:** Samples (taken at Edinburgh airport wastewater treatment plant inlet point) spiked with the Wuhan strain and the Alpha variant for SARS-CoV-2 for the study (gc: Genome copies).

Sample code	Virus spiking type and approx. concentration	Number of replicates
EDI-neat1	None	3
EDI-neat2	None	3
EDI-Wuhan_low	Wuhan strain: 10^4^ gc/ml	3
EDI-Wuhan_high	Wuhan strain: 10^5^ gc/ml	3
EDI-Wuhan_high-Alpha_low	Wuhan strain: 10^5^ gc/ml	3
	Alpha variant: 10^4^ gc/ml	
EDI-Wuhan_low-Alpha_high	Wuhan strain: 10^4^ gc/ml	3
	Alpha variant: 10^5^ gc/ml	

#### 2.1.2 Set 2—Surveillance samples

The virus concentration method using beef extract elution and polyethylene glycol (PEG) precipitation (see Section 2.2 for details) that recovered most of the spiked viruses was applied to the processing of the further samples taken at Edinburgh, Heathrow and Bristol airports between 8^th^ and 31^st^ March 2022 (n = 180; Table 2). At Edinburgh airport, samples were taken from manholes to capture the outflow of the international terminal (JR1-3; approx. location 55°56’50.7"N 3°21’45.1"W), from a pumping station (P1; approx. location 55°56’39.7"N 3°22’23.3"W) where vacuum trucks used to collect wastewater from aircraft deposit their contents, and at the WWTP (approx. location 55°56’36.6"N 3°23’55.8"W) where all sewage from the airport is discharged. At Heathrow airport, samples were taken from a manhole at the Central Terminal Area (CTA; approx. location 51°28’22.2"N 0°27’03.4"W) capturing terminals T1, T2, and T3. Ten additional samples were also taken directly from the vacuum trucks, which collected aircraft wastewater directly from ten individual aircraft during the day of sampling. At Bristol airport, samples were taken from manholes to sewers collecting wastewater predominantly serving the arrival terminal (MH2; approx. location 51°23’10.6"N 2°42’53.0"W) and from the entire airport (MH3; approx. location 51°23’15.4"N 2°42’20.5"W). Additional samples were taken from a deposit sites where vacuum trucks collecting aircraft wastewater unload their content using an autosampler programmed to take grab samples during each wastewater discharge event (MH1; approx. location 51°23’09.5"N 2°42’13.4"W). These samples contained a mixture of wastewater originating from 4–17 planes (S1 Table). At each location, 24-hour composite samples were taken using autosamplers, except when samples were taken from vacuum trucks at Heathrow airport. In that case grab samples were taken using a clean wastewater sampling device.

**Table 2:** Sampling sites, sample types, numbers, and physico-chemical properties for wastewater collected at three UK airports.

Sampling site	Sampling period	n	Sample type	pH	Turbidity NTU	Conductivity μS/cm	Ammonium mg N/l	Orthophosphate mg P/l
EDI-JR1	8–23 March 2022	44	2-hour composite	7.79 (0.07)	7.28 (2.02)	1038 (196)	27.7 (1.83)	5.5 (1.25)
EDI-JR2	8–23 March 2022	16	24-hour composite	7.44 (0.12)	85.7 (9.1)	1576 (206)	71.6 (6.54)	11.0 (5.25)
EDI-JR3	8–19 March 2022	8	24-hour composite	7.64 (0.09)	92.2 (30.9)	1330 (81)	81.9 (13.49)	8.9 (3.11)
EDI-P1	8–23 March 2022	15	12-hour composite	7.54 (0.11)	54.5 (10.6)	1547 (103)	67.2 (8.79)	5.9 (1.71)
EDI-WWTP	8–24 March 2022	13	24-hour composite	7.32 (0.02)	34.3 (16.1)	844 (22)	20.7 (4.03)	6.3 (2.60)
LHR-CTA wet well	8–24 March 2022	15	24-hour composite	7.59 (0.08)	202 (40.2)	3169 (212)	129.2 (6.91)	24.0 (5.71)
LHR-Vacuum truck	16 March 2022	10	Grab	8.84 (0.13)		1205 (989)	88.8 (11.98)	109.7 (14.22)
BRS-MH2	21–28 March 2022	24	21-hour composite	8.77 (0.05)	366 (103)	2498 (295)	120.9 (5.31)	8.3 (1.16)
BRS-MH3	21–29 March 2022	28	21-hour composite	8.06 (0.08)	497 (157)	2026 (111)	106.5 (5.27)	11.6 (1.30)
BRS-MH1 Vacuum truck	25–28 March 2022	7	Grab	8.78 (0.29)	1410 (354)	12718 (3060)	83.7 (13.90)	93.7 (23.30)

Where applicable, numbers represent mean values with the standard error shown in brackets. EDI: Edinburgh airport; LHR: London Heathrow airport; BRS: Bristol airport; NTU: nephelometric turbidity unit. Due to sample availability, some samples were not tested for physico-chemical properties.

The sample pH, turbidity, electrical conductivity, ammonium, and orthophosphate ion concentrations were measured as described previously [8]. Formaldehyde was measured in the samples derived from aircrafts using a Formaldehyde Assay Kit (Abcam, UK) following the manufacturer’s instructions. Total chorine was measured in the aircraft wastewater samples taken at Heathrow and Bristol samples after centrifugation (10,000 x *g*, 10 min) using a total chlorine meter (Hanna Instruments, USA) according to the manufacturer’s instructions.

### 2.2 Sample process for virus concentration

We trialled five sample concentration methods on the samples spiked with SARS-CoV-2 (Table 1) and used the best performing method on the samples taken for surveillance (Table 2).

**Fig 1:**
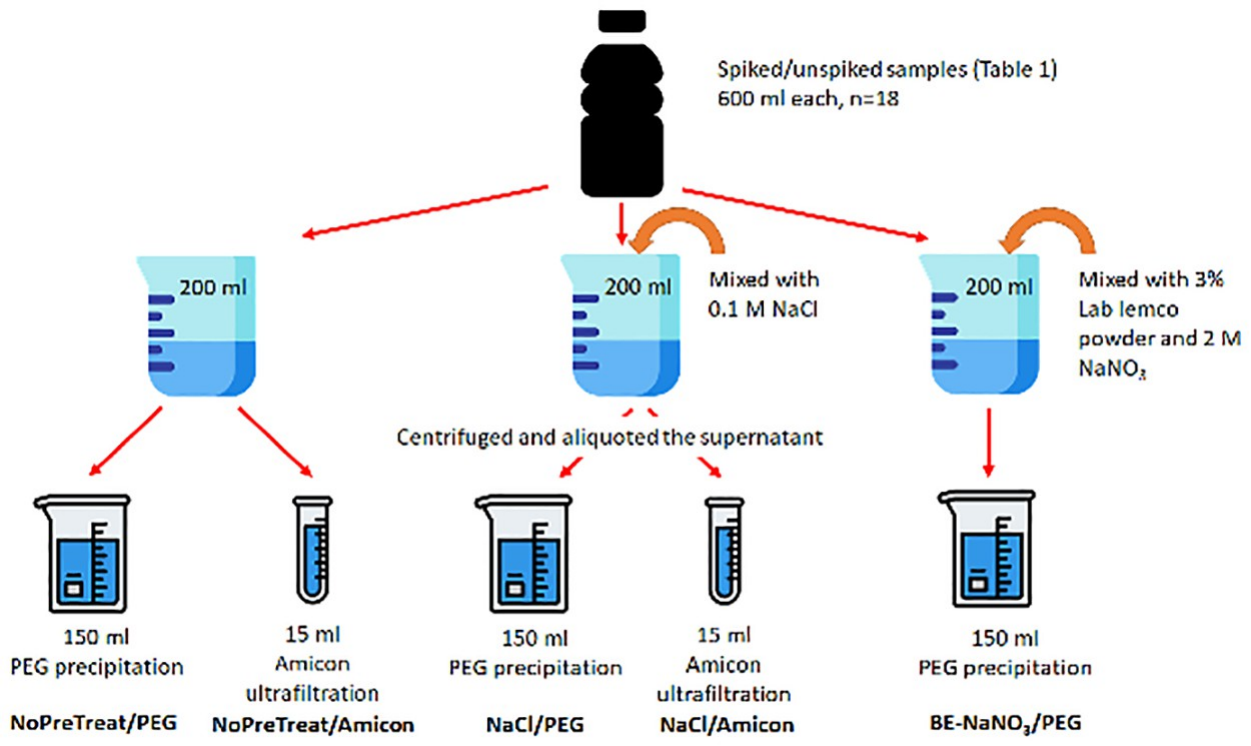
Sample pre-treatment and concentration methods used on the wastewater samples. PEG: polyethylene glycol.

The samples spiked with SARS-CoV-2 (Table 1) were divided into three 200-ml aliquot sets and were spiked with approximately 10^5^ gc phi6 bacteriophage as a process control [32]. Process positive controls (water spiked with phi6) and process negative controls (water only) were also concentrated along with each batch of samples to investigate recovery efficiency and cross-contamination. We used two pre-treatments (sodium chloride and beef extract-sodium nitrate) and two types of concentration (polyethylene glycol (PEG) precipitation and ultrafiltration) on each sample (Fig 1).

One set of the 200-ml aliquots was not pre-treated, being only concentrated using PEG precipitation and ultrafiltration, as described elsewhere [33]. The samples were centrifugated at 10,000 x *g* at 4°C for 10 min. Then, 15 ml of the supernatant was transferred to a 10 kDa Amicon Ultra-15 centrifugal filters (Merck Life Science UK Ltd, UK). The samples were centrifuged at 5,000 x *g* for 30–60 min to reach a final volume of 200–500 μl, resulting in NoPreTreat/Amicon samples. The filtrates were discarded. For NoPreTreat/PEG samples, another 150 ml of the primary supernatant was also separated, and the pH was adjusted to 7.0–7.5 and mixed with PEG8000 and NaCl to reach final concentrations of 10% and 2%, respectively, and incubated at 4°C for 16 hours. The mixture was centrifuged at 10,000 x *g* at 4°C for 30 min. The resulting pellet was then subjected to RNA/DNA extraction.

The second set of samples were mixed with NaCl to reach a final concentration of 0.1 M. The mixtures were incubated at 50 rev min^-1^ at room temperature for 30 min. Then, the samples were centrifuged to clarify solid matter, from which 150 ml of the supernatant was PEG-precipitated (NaCl/PEG) and 15 ml was ultrafiltered (NaCl/Amicon), as described above.

The third set of samples were mixed with Lab Lemco beef extract (Oxoid, USA) and sodium nitrate to reach the final concentration of 3% and 2 M, respectively [33]. After the pH of the mixtures were adjusted to 3.5–5.5, the solutions were incubated at 50 rev min^-1^ at room temperature for 30 min. Subsequently, the samples were centrifuged and PEG-precipitated, as described above (resulting in BE-NaNO_3_/PEG samples). As this method performed the best (see Section 3.1 for details), this approach was used on the surveillance samples (Table 2).

### 2.3 Viral RNA/DNA extraction

The viral nucleic acids were extracted from the PEG pellets and the ultrafiltered concentrate using the NucliSens extraction kit (BioMerieux, France) on a Kingfisher 96 Flex system (Thermo Scientific, USA) as described previously [32, 34]. The final volume of the eluent was 100 μl. To assess extraction performance and cross-contamination, extraction positive (deionised water spiked with phi6) and negative controls (distilled water) were used.

### 2.4 Viral RNA/DNA quantification

We quantified SARS-CoV-2 and phi6 RNA using a duplex RT-qPCR and crAssphage using a qPCR assay on a QuantStudio Flex 6 real-time PCR machine (Applied Biosystems Inc., USA) as previously described [32, 33]. Each assay contained a dilution series of standards of known concentrations [33] and non-template controls, which were negative in each reaction. Assay details and performance are summarised in Table 3.

**Table 3:** RT-qPCR and qPCR assay details and performance for the viral targets.

	Oligo reference	Assay	Standard curve		
			Slope	R^2^	Efficiency %
SARS-CoV-2 (N1)	[35]	TaqMan Viral 1-step RT-qPCR master mix (Applied Biosystems Inc., USA)	-3.51 –-3.21	0.993–0.999	92.6–104.9
Phi6	[36]		-3.55 –-3.11	0.969–0.999	91.4–109.9
CrAssphage	[37]	QuantiNova Probe qPCR mix (Qiagen, Germany)	-3.41 –-3.18	0.994–0.998	96.3–106.3

### 2.5 Data analysis

The preliminary qPCR data analysis and quality control was performed using the QuantStudio Flex 6 real-time PCR software v1.7.1 (Applied Biosystems Inc., USA). The viral concentrations were expressed as gc/μl RNA/DNA extract. The viral concentrations (gc/ml) in the concentrated samples were calculated as:

$$\frac{\text{virus}\;\text{concentration}\;\text{of}\;\text{the}\;\text{nucleic}\;\text{acid}\;\text{extract}\times \text{extract}\;\text{volume}}{\text{volume}\;\text{of}\;\text{sample}\;\text{supernatant}\;\text{processed}}$$ (1)

For the surveillance samples, concentrations were interpreted as gc/l wastewater. Virus recoveries for the phi6 process control virus were calculated as:

$$\frac{\text{Phi}6\;\text{concentration}\;\text{of}\;\text{the}\;\text{concentrated}\;\text{samples}}{\text{Phi}6\;\text{concentration}\;\text{of}\;\text{the}\;\text{unconcentrated}\;\text{samples}}\;\times 100\%$$ (2)

Subsequent data analysis and statistical tests were carried out in R version 4.1.2 [38], utilising packages “readxl”, “tidyr”, “dplyr”, “tidyverse”, and “ggplot2”. We tested for significant differences in viral concentration (gc/ml) across methods using a Kruskal-Wallis rank sum test and Pairwise Wilcoxon Tests. Spearman correlation was used to assess correlation between chemical and viral data.

Metadata can be found in S2 Table.

## 3. Results

### 3.1 Wastewater concentration method selection

Of the five methods tested, BE-NaNO_3_/PEG precipitation method showed the highest virus recovery in comparison to the other methods, both for the SARS-CoV-2 N1 gene fragment and for the phi6 process control virus (Table 4).

**Table 4:** Summary statistics for SARS-CoV-2 (N1 gene fragment) and phi6 showing higher viral recovery using BE-NaNO_3_ pre-treatment.

Virus	Method	Mean concentration (gc/ml)	SD	Median concentration (gc/ml)	IQR
SARS-CoV-2	BE-NaNO_3_/PEG	1858	2629	943	1375
	NaCl/PEG	1227	2094	347	1450
	NaCl/Amicon	655	877	248	760
	NoPreTreat/PEG	433	442	192	838
	NoPreTreat/Amicon	858	1208	283	959
Phi6	BE-NaNO_3_/PEG	4193	1706	4094	2891
	NaCl/PEG	58	96	13	52
	NaCl/Amicon	15	28	4	5
	NoPreTreat/PEG	8	6	8	12
	NoPreTreat/Amicon	28	32	13	38
CrAssphage	BE-NaNO_3_/PEG	16707	12875	15202	19972
	NaCl/PEG	12698	9775	13844	16730
	NaCl/Amicon	5280	857	5453	1241
	NoPreTreat/PEG	5237	7275	1134	4695
	NoPreTreat/Amicon	3124	3124	3031	1076

gc: genome copies; SD: standard deviation; IQR: interquartile range.

**Fig 2:**
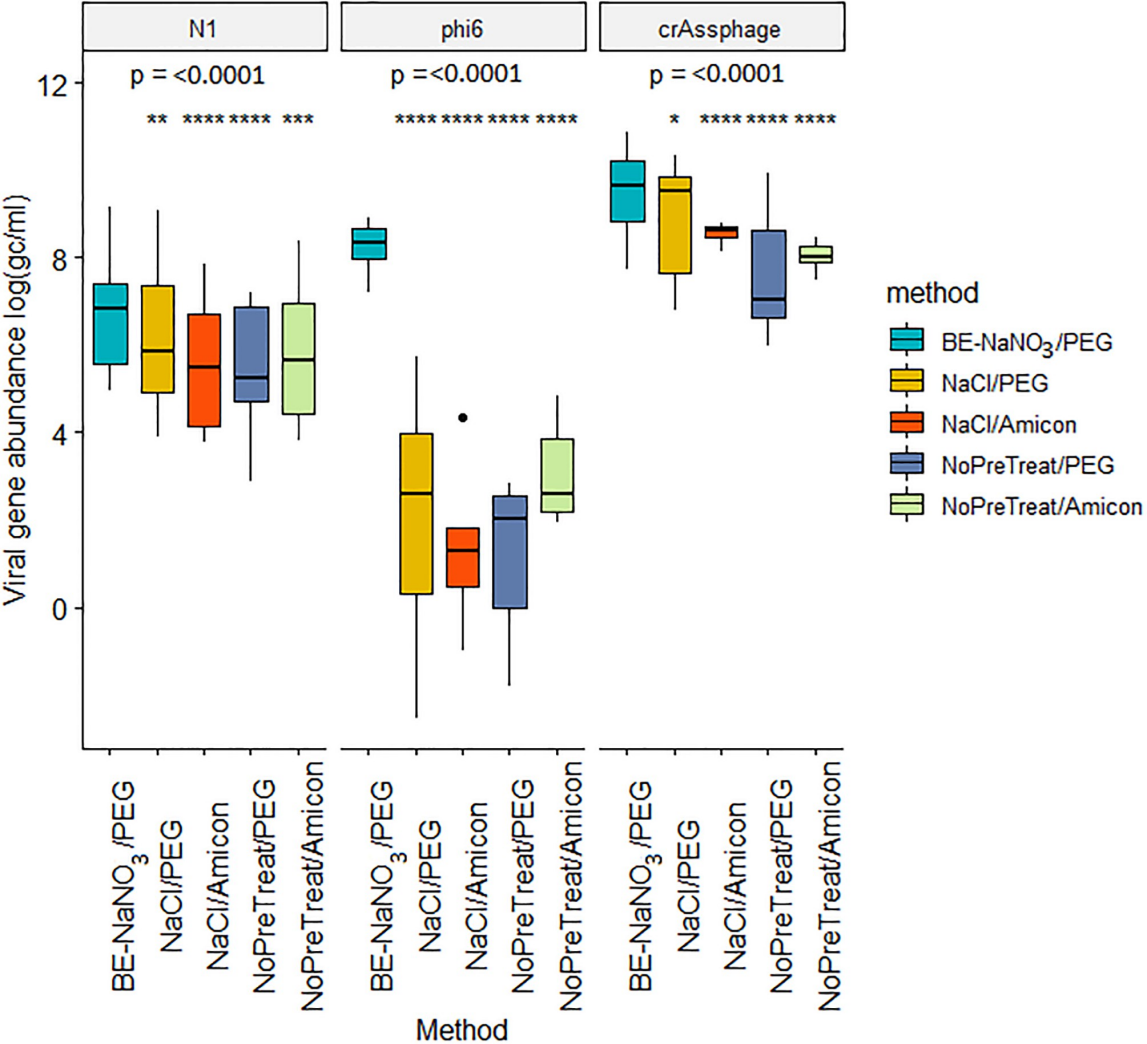
Boxplots showing the viral gene abundance recovered for N1, phi6 and crAssphage with different processing methods of spiked wastewater. Global p-value is shown for each target, and significance codes (≤0.0001 ‘****’, ≤0.001 ‘***’, ≤0.01 ‘**’, ≤0.05 ‘*’) indicate statistically significant difference in results of BE-NaNO_3_/PEG methods compared to each of the remaining methods.

A Shapiro-Wilk normality test showed that the distribution of the data departed significantly from normality for SARS-CoV-2 (W = 0.76, p<0.01), Phi6 (W = 0.57, p<0.01) and crAssphage (W = 0.76, p<0.01). The BE-NaNO_3_ method was significantly better than the other four methods for SARS-CoV-2, phi6 recovery and crAssphage (Fig 2) using a Kruskal Wallis rank sum test followed by Pairwise Wilcoxon Tests. The surveillance samples were therefore processed using a BE-NaNO_3_ pre-treatment and PEG precipitation.

### 3.2 Surveillance of SARS-CoV-2 in wastewater at airports

The wastewater samples were spiked with phi6 process control virus prior to concentration to assess viral recovery efficiency. Overall, the control virus was recovered in most samples, however, no virus recovery was noted in the samples derived from vacuum trucks at Heathrow airport (Table 5). The recovery rates were also low for samples taken from vacuum trucks at the Bristol site (33%) and at the Heathrow CTA site (50%). In many cases, crAssphage and SARS-CoV-2 were detected in samples where no Phi6 recovery probably due to low spiking concentrations.

**Table 5:** SARS-CoV-2 and crAssphage detection rates and concentrations along with Phi6 process control recoveries for airport wastewater surveillance.

Sampling site	SARS-CoV-2 (N1)		crAssphage		Phi6	
	Positivity rate	Concentration gc/l	Positivity rate	Concentration gc/l	Positivity rate	Recovery %
EDI-JR1	75%	3.1x10^3^ (9.7x10^2^)	100%	5.3x10^6^ (4.7x10^6^)	100%	47.6 (4.3)
EDI-JR2	100%	8.9x10^4^ (2.1x10^4^)	100%	4.9x10^7^ (3.6x10^7^)	100%	19.7 (4.6)
EDI-JR3	100%	3.9x10^5^(2.7x10^5^)	100%	2.6x10^7^ (1.5x10^7^)	100%	41.7 (18.7)
EDI-P1	100%	6.7x10^4^ (1.3x10^4^)	100%	1.8x10^7^ (7.5x10^6^)	64%	53.3 (27.7)
EDI-WWTP	92%	2.4x10^5^ (1.2x10^5^)	100%	2.9x10^7^ (1.6x10^7^)	82%	13.1 (3.2)
LHR-CTA wet well	100%	8.2x10^4^ (1.5x10^4^)	100%	3.1x10^7^ (5.8x10^6^)	50%	27.0 (12.1)
LHR-VT	80%	1.6x10^6^ (8.9x10^5^)	40%	5.6x10^7^ (1.8x10^7^)	0%	-
BRS-MH2	100%	1.8x10^5^ (4.4x10^4^)	100%	2.3x10^7^ (7.3x10^6^)	75%	57.8 (8.0)
BRS-MH3	100%	4.7x10^5^ (1.6x10^5^)	100%	5.3x10^7^ (2.0x10^7^)	79%	23.4 (6.1)
BRS-MH1 VT	100%	4.0x10^6^ (2.3x10^6^)	100%	7.4x10^7^ (5.0x10^7^)	33%	44.8 (34.7)

Where applicable, numbers represent mean values with the standard error shown in brackets. gc: genome copies. Due to sample availability, some samples were not tested for crAssphage. VT–Vacuum Truck.

**Fig 3:**
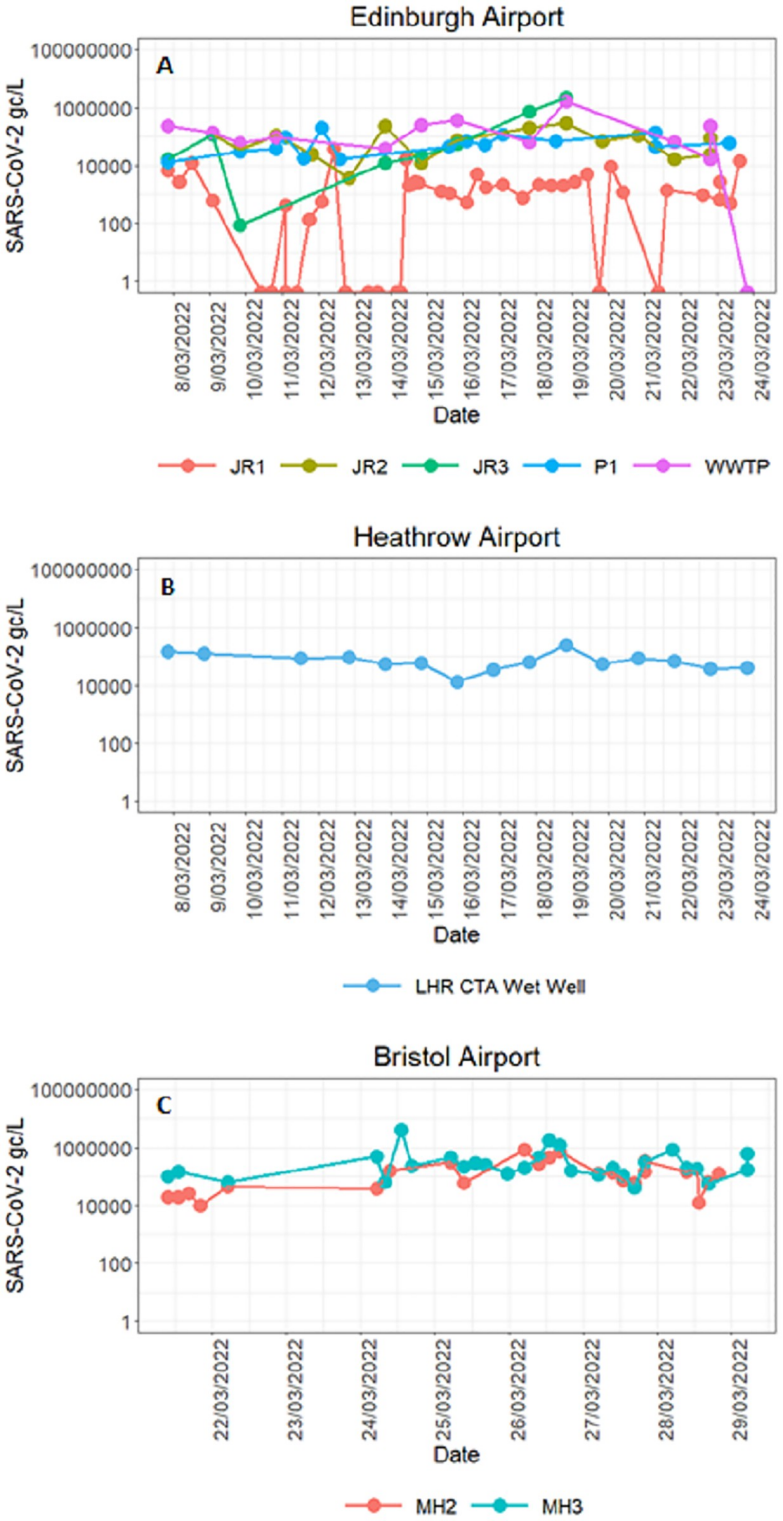
SARS-CoV-2 RNA concentrations in genome copies (gc)/l at (A) Edinburgh, (B) Heathrow and (C) Bristol airports.

The majority of the wastewater samples taken at the three airports were positive for SARS-CoV-2 RNA and crAssphage DNA. The lowest SARS-CoV-2 concentration and detection rates were observed in the JR1 Edinburgh airport samples (Table 5). The remaining samples taken at Edinburgh were all positive, except for one sample taken at the WWTP, with recorded concentrations of between 1x10^2^ and 2x10^6^ gc/l. All samples taken at Edinburgh airport contained crAssphage at high concentrations (Table 5). The sample pH and orthophosphate levels showed little variation among the sampling sites, however, considerably higher turbidity, electrical conductivity and ammonium levels were noted at JR2, JR3, and P1 sites compared to JR1 and WWTP samples (Table 2). No significant trends in SARS-CoV-2 levels over time were observed at any of the sampling sites (Fig 3).

At Heathrow and Bristol airports, all sewage samples derived from the terminals were positive for SARS-CoV-2 and crAssphage, with concentrations in the range of 1x10^4^ − 4x10^6^ gc/l and 2x10^7^ − 6x10^7^ gc/l, respectively (Table 5; Fig 3). At these sites the ammonium and turbidity levels in the samples were higher than those observed in samples collected at the Edinburgh sites. At Bristol sites, the sample pH levels were also higher than the pH in samples collected at the other two locations (Table 5). No correlation between viral concentrations and chemical data was found.

**Fig 4:**
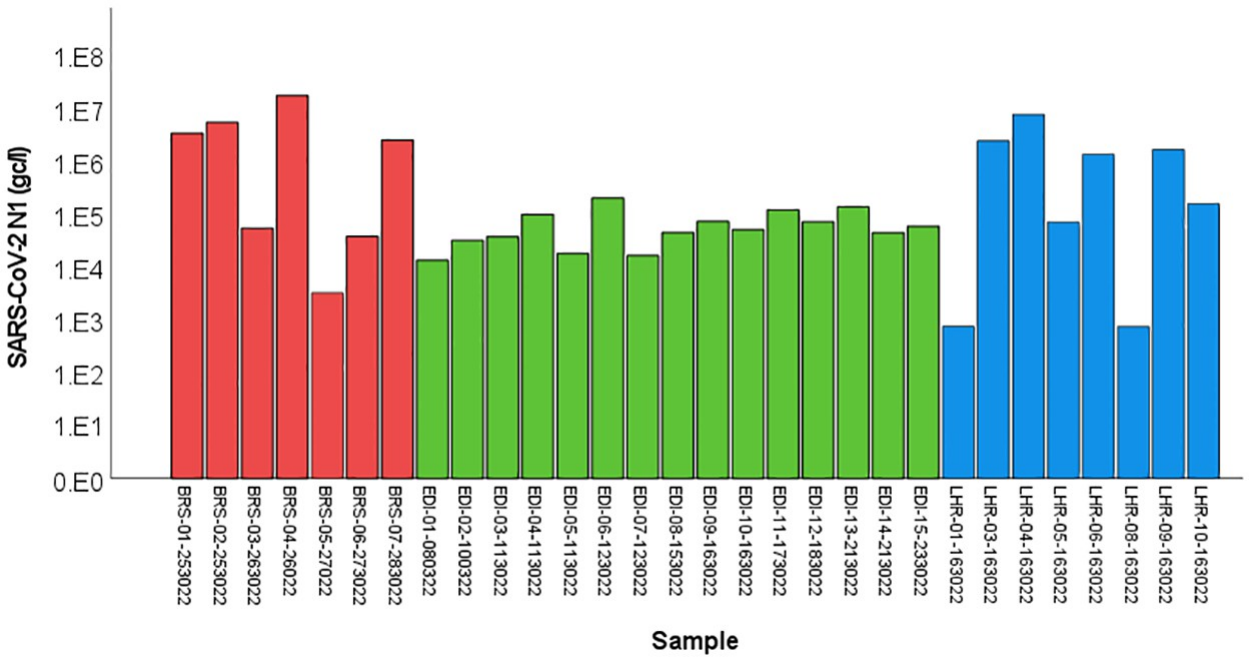
SARS-CoV-2 RNA concentration in samples taken from vacuum trucks at Bristol (BRS–red bars), at Edinburgh (EDI–green bars) and at Heathrow (LHR–blue bars) airports.

The content of vacuum trucks carrying sewage derived from aircraft were also tested for SARS-CoV-2. All samples taken at Edinburgh and Bristol sites were positive for SARS-CoV-2 and all but two samples taken at Heathrow returned positive (Fig 4). CrAssphage was detected in all samples taken from vacuum trucks at Edinburgh and Bristol airports and 40% of the Heathrow vacuum truck samples were also positive for that virus. No correlation between the crAssphage and SARS-CoV-2 detection rates or concentrations were identified. Sample pH, turbidity, orthophosphate levels and, in some cases, electrical conductivity were also notably higher than those levels in the rest of the samples (Table 5). In order to assess the presence of biocides often used for aircraft wastewater treatment, we tested the samples for the presence of formaldehyde and chlorine. The concentration of formaldehyde in the samples was negligible (<0.5mg/l) and chlorine was not detected either. Due to the viscosity of the samples taken from the vacuum truck deposit site at Heathrow airport, turbidity could not be measured.

## 4. Discussion

International air travel has had a significant impact on the rapid spread of COVID-19 and greatly contributed to the development of the pandemic. It is now established that travellers arriving from mainland Europe to the UK in early 2020 were responsible for introducing *ca*. 1300 SARS-CoV-2 lineages, resulting in the first wave of the pandemic [39]. Even with significant non-pharmaceutical interventions being in place (i.e., social distancing, face covering, negative COVID test results prior to travel), in-flight transmission continued to occur with an attack rate (i.e. the rate of infected individuals in a population at risk) of 10–17% [40]. Due to the successful roll-out of vaccination programmes worldwide, COVID-19 restrictions have been lifted in many airports and during flights. While vaccination reduces the number of COVID-19-related hospitalisations and deaths, it has limited capacity to reduce the spread of infections and to contain outbreaks [41]. With continued transmission, which occurs more rapidly in confined spaces [42], such as aircraft, the introduction of novel lineages via air travel is a significant risk. Besides the clinical surveillance of passengers, research on alternative approaches to reduce transmission in confined spaces, such as updating air circulation systems onboard flights, is a priority.

In this study, we assessed the usefulness of WBE for the quantitative assessment of SARS-CoV-2 in wastewater arrival locations at three international airports in the UK. The samples were collected from i) sewers connected to the arrival halls in the airport terminals, ii) vacuum trucks and deposit sites where vacuum trucks collecting aircraft wastewater unload their contents, and iii) a wastewater treatment plant receiving wastewater serving the airport. We noted considerable differences in the solids content and other physico-chemical properties of wastewater taken from the sewers and aircraft in comparison to municipal wastewater (Table 2). Hence, we subsequently trialled different approaches to enhance viral nucleic acid recovery and provide consistent results from highly turbid samples.

Most virus concentration methods applied to wastewater samples begin with the exclusion of solid matter via filtration or centrifugation. However, previous research suggests that up to 25% of SARS-CoV-2 RNA may be retained in the solid matrix of municipal wastewater [22, 23] and this ratio may be even higher in highly turbid samples. Therefore, in this study we trialled pre-treatment methods to detach viruses and viral nucleic acids from solids and found that the application of beef extract significantly increases recovery compared to the application of salt or no treatment (Fig 2). Beef extract has been shown to enhance detachment of viruses and nucleic acids from solid matter in wastewater and environmental samples, while at the same time binding viral particles, which assists their precipitation [33, 43–45]. However, when centrifugation is used to clarify the sample after beef extract treatment, the solution is not suitable for subsequent ultrafiltration due to the remaining proteins clogging the filter, further increasing the processing time.

The application of NaCl also enhanced viral recovery compared to samples where no pre-treatment was applied, potentially through the detachment of viral particles from solid particles. It can be explained by an additional competition created by Na^+^ and Cl^-^ ions for the polar binding sites on the surface of solid particles, which usually comprises organic matter. NaCl treatment did not require pH adjustment and the resulting samples were suitable for ultrafiltration and, hence, it may be applied for WBE when sample processing is a time-limiting step.

After the samples (with or without pre-treatment) were clarified using centrifugation, the resulting supernatants were concentrated using PEG precipitation or ultrafiltration. When no pre-treatment was applied, the ultrafiltration-based concentration performed similar or better than PEG precipitation, as shown previously [30, 33]. When salt pre-treatment was used, the PEG precipitation gave slightly higher viral recoveries than ultrafiltration (Fig 2). The best performing method was the beef extract-based pre-treatment coupled with PEG precipitation, which enabled the consistent virus concentration in 150 ml samples and, hence, this method was used for subsequent SARS-CoV-2 monitoring.

Using the BE-NaNO_3_ concentration method, we successfully recovered SARS-CoV-2 RNA and the DNA of the faecal indicator virus, crAssphage. However, we noted that the process control virus (phi6) used to assess viral recovery was less frequently detected in the samples than SARS-CoV-2 and crAssphage, especially in the samples derived from vacuum trucks. Previous studies also noted lower recoveries for phi6 than for coronaviruses in concentrated wastewater [46, 47], suggesting that phi6 bacteriophage may not be a useful indicator for SARS-CoV-2 recovery in all types of wastewater samples. This may be due to the decreased genetic stability of phi6 in comparison to coronaviruses or crAssphage when exposed to complex wastewater matrices. Nonetheless, the phi6 virus may be used as a process control for less challenging sample matrices, such as municipal wastewater [16, 32, 48].

Our results suggest that the highest SARS-CoV-2 concentration and detection rates may be achieved when samples collected close to source are analysed. In wastewater samples taken from sewers close to the airport terminals, for almost all cases SARS-CoV-2 was detected at high concentrations, whereas samples further away from the source and at the receiving wastewater treatment plant demonstrated lower detection rates. This may be due to viral decay and/or accumulation in biofilm in the sewers [49, 50].

All but two samples derived from aircraft were also positive for SARS-CoV-2, and all those collected from airplanes landing at Bristol airport were SARS-CoV-2 positive. However, as those samples contained wastewater from several aircraft, the origin of the viruses could not be determined. Similarly, at Edinburgh airport, where all aircraft-derived samples were positive for SARS-CoV-2, it was not possible to determine the flight origin as the samples were taken from a deposit site, where cross-contamination is inevitable. Whole genome sequencing may also be useful to determine the origin of novel strains [51], but these approaches may be hard to implement for difficult matrices, such as aircraft wastewater. One of the two samples from aircraft landing at Heathrow airport that tested negative for SARS-CoV-2 were also negative for crAssphage, suggesting that the wastewater may have contained disinfectants and other cleaning agents [52], indicated by unusual colour and pH, which may degrade viruses and viral RNA. Hence, it is possible that the wastewater contained SARS-CoV-2 before such treatment but is unrecoverable after chemical treatment. More detailed studies are needed to assess viral degradation and recovery under such conditions [53].

The high SARS-CoV-2 detection frequencies in wastewater from terminal sewers and aircraft suggest that passengers arriving at the airports and/or staff members working at the terminals had ongoing COVID-19 infections. The COVID-19 restrictions were lifted in England on the 18^th^ March 2022, and no differences in the concentrations of SARS-CoV-2 in wastewater before and after that date were noted. This finding may suggest that the travel restrictions, such as negative COVID-19 tests, social distancing and mask wearing, were not suitable to filter asymptomatic and pre-symptomatic individual. Previous studies have found the effectiveness of these travel restrictions depends on early intervention, enforcement and compliance, and the sensitivity of screening tests [54, 55].

Overall, our results suggest that WBE may be used as a simple monitoring tool for SARS-CoV-2 and other viral diseases at airports and aircraft, to identify outbreak hotspots internationally and to observe trends in the infection prevalence [19, 20], however, the approach has limitations, which must be considered. Cross-contamination may occur as one vacuum truck is used for emptying sewage from several aircraft with no cleaning in between planes. In order to avoid that, sampling from aircraft may be a viable option, however, considering the number of flights arriving at international airports daily, that may not be feasible due to logistics issues and high costs. Furthermore, sampling of aircraft may be biased due to in-flight toilet usage and behaviour trends. A recent study assessing toilet use on short- and long-haul flights, discovered that aircraft wastewater probably captures 8–14% of SARS-CoV-2 cases [56]. The same study estimated that approximately 62% of the long-haul flights would have wastewater containing SARS-CoV-2, which is a slightly lower prevalence that observed here (80–100%). The lower estimate in the former study is most likely due to underreporting of defecation habits when undertaking social behaviour surveys, uncertainties in the proportion of individuals that shed SARS-CoV-2 in faeces, and the lack of consideration for capture of other bodily fluids in aircraft toilet wastewater (e.g. saliva) [56].

Even with highly effective extraction methods, wastewater samples derived from aircraft may contain solid matter and additives which reduce the chances of successful virus detection [53]. Monitoring sewage from airport terminals is less challenging, but also less informative on the origin of the pathogen due to the high number of people contributing to a sample. In some cases this wastewater may capture both inbound and outbound passengers as well as office staff working in the same terminal. Nonetheless, the regular sampling of airport and aircraft wastewater can be useful as targeted monitoring system for emerging diseases and other agents (e.g. anti-microbial resistance genes) that have not yet become endemic in the country. Using qPCR or digital PCR-based quantitative analysis of novel viral agents would enable rapid assessment and also help shortlisting samples for further analysis, e.g. whole genome sequencing and metagenomics, enabling deeper understanding on the pathogens circulating globally.

## Supporting information

S1 Table Details on the aircraft that emptied their wastewater in manhole 1 (MH1) at Bristol airport during each sampling periods. (DOCX)

S2 Table Analysis results. (DOCX)
