# Supplementary material for: Wastewater-based monitoring of SARS-CoV-2 at UK airports and its potential role in international public health surveillance
Source: PLOS Glob Public Health. 2023 Jan 19;3(1):e0001346. doi: 10.1371/journal.pgph.0001346 (PMC10021541; doi:10.1371/journal.pgph.0001346)
Supplement: S1 Table — (DOCX) [file pgph.0001346.s001.docx]

S1 Table. Details on the aircraft that emptied their wastewater in manhole 1 (MH1) at Bristol airport during each sampling periods

| **Collection date** | ***Number of flights*** | **Flight numbers / Port of origin** |
| --- | --- | --- |
| 26/03/2022 | 4 | TFS/AYT/TFS/HRG |
| 26/03/2022 | 4 | TFS/INN/PFO/ACE |
| 26/03/2022 | 17 | EZY FLIGTS: 6296, 424, 408, 6168, 6224, 6160, 571, 448, 430, 6050, 6238, 6074, 6096  FR FLIGHT: 508, 4756, 4441, 8122 |
| 27/03/2022 | 8 | EZY FLIGHTS: 6148, 6104, 6292, 6072, 6114, 6066, 6194 FR FLIGHT: 4754 |
| 28/03/2022 | 17 | EZY FLIGHTS: 6236, 6256, 6156, 6158, 6152, 6160, 6258, 6196, 6278, 6246, 6096, 6262, 6020, 6294  FR FLIGHTS: 8297, 4758, 7513 |
| 28/03/2022 | 12 | EZY FLIGHTS: 6190, 6206, 6052, 6072, 6040, 6096, 446, 6013, 410, 394, 6254  FR FLIGHTS: 8241 |
| 29/03/2022 | N/A | N/A |
