# Supplementary material for: Wastewater-based monitoring of SARS-CoV-2 at UK airports and its potential role in international public health surveillance
Source: PLOS Glob Public Health. 2023 Jan 19;3(1):e0001346. doi: 10.1371/journal.pgph.0001346 (PMC10021541; doi:10.1371/journal.pgph.0001346)
Supplement: S2 Table — (DOCX) [file pgph.0001346.s002.docx]

S2 Table. Analysis results

| Airport | Site | Sample collected | Sample type | SARS-CoV-2 gc/l | pH | Conductivity us/cm | Ammonium mg/l | Orthophosphate mg/l | CrAssphage gc/l | Turbidity NTU | Phi6 Rec% |
| --- | --- | --- | --- | --- | --- | --- | --- | --- | --- | --- | --- |
| Edinburgh | JR1 | 08032022:0001 | Composite | 6633 | 7.87 | 1132 | 19.75 | 1.35 | 56329680 | 1.4 |  |
| Edinburgh | JR1 | 08032022:0800 | Composite | 2813 | 7.87 | 939.2 | 39.31 | 2.43 | 10025103 | 50 |  |
| Edinburgh | JR1 | 08032022:1600 | Composite | 11880 | 7.54 | 871.2 | 34.02 | 2.46 | 14318393 | 44.4 |  |
| Edinburgh | JR1 | 09032022:0600 | Composite | 600 | 7.29 | 808.6 | 16.69 | 1.37 | 22446167 | 7.8 |  |
| Edinburgh | JR1 | 10032022:1400 | Composite |  | 7.53 | 900.3 | 19.57 | 0.84 |  | 5.2 | 40.58 |
| Edinburgh | JR1 | 10032022:2100 | Composite |  | 7.57 | 857.4 | 21.24 | 0.87 |  | 1.9 | 61.27 |
| Edinburgh | JR1 | 11032022:0600 | Composite | 420 | 7.70 | 1097 | 20.61 | 1.76 |  | 2.4 | 97.20 |
| Edinburgh | JR1 | 11032022:0600 | Composite |  | 7.85 | 907.1 | 9.76 | 0.23 |  | 1.5 | 83.69 |
| Edinburgh | JR1 | 11032022:1400 | Composite |  | 8.14 | 97.1 | 40.56 | 1.90 |  | 2.6 | 42.55 |
| Edinburgh | JR1 | 11032022:2200 | Composite | 133 | 7.42 | 650.5 | 16.48 | 0.55 |  | 6 | 81.34 |
| Edinburgh | JR1 | 12032022:0600 | Composite | 567 | 8.34 | 1489 | 51.18 | 3.10 |  | 3.3 | 78.64 |
| Edinburgh | JR1 | 12032022:1400 | Composite | 36980 | 8.14 | 1008 | 36.81 | 1.15 |  | 2.7 | 42.13 |
| Edinburgh | JR1 | 12032022:2200 | Composite |  | 7.76 | 8715 | 19.08 | 0.76 |  | 7.6 | 39.05 |
| Edinburgh | JR1 | 13032022:1300 | Composite |  | 7.59 | 930.8 | 6.40 | 1.86 |  | 3.2 | 14.05 |
| Edinburgh | JR1 | 13032022:1900 | Composite |  | 7.90 | 730.3 | 16.99 | 1.09 |  | 2.8 | 44.57 |
| Edinburgh | JR1 | 14032022:0800 | Composite |  | 8.39 | 910.7 | 57.46 | 4.03 | 1128280 |  | 44.44 |
| Edinburgh | JR1 | 14032022:1000 | Composite |  | 7.73 | 580.9 | 23.77 | 0.84 | 5396423 |  | 28.46 |
| Edinburgh | JR1 | 14032022:1400 | Composite | 16587 | 8.39 | 893 | 46.57 | 2.56 | 866770 |  | 42.90 |
| Edinburgh | JR1 | 14032022:1600 | Composite | 2073 | 7.68 | 637.6 | 20.61 | 0.98 | 984116.7 |  | 32.98 |
| Edinburgh | JR1 | 14032022:1800 | Composite | 2227 | 7.94 | 637.6 | 28.68 | 1.45 | 2599003 |  | 69.70 |
| Edinburgh | JR1 | 14032022:2000 | Composite | 2753 | 6.44 | 844.5 | 43.74 | 2.66 | 900000 |  | 57.74 |
| Edinburgh | JR1 | 14032022:2200 | Composite | 2533 | 7.85 | 875.9 | 37.30 | 0.29 | 1745517 |  | 61.14 |
| Edinburgh | JR1 | 15032022:1300 | Composite | 1313 | 7.44 | 685.7 | 28.69 | 2.84 | 767136.7 |  | 22.56 |
| Edinburgh | JR1 | 15032022:1900 | Composite | 1127 | 7.63 | 730.5 | 31.63 | 3.36 | 7873133 |  | 50.87 |
| Edinburgh | JR1 | 16032022:0600 | Composite | 507 | 7.39 | 709.5 | 47.43 | 4.22 | 867120 |  | 18.68 |
| Edinburgh | JR1 | 16032022:1300 | Composite | 4840 |  |  |  |  | 1465197 |  | 24.33 |
| Edinburgh | JR1 | 16032022:1900 | Composite | 1753 |  |  |  |  | 1148910 |  | 77.95 |
| Edinburgh | JR1 | 17032022:0600 | Composite | 2260 |  |  |  |  | 2011330 |  | 100.00 |
| Edinburgh | JR1 | 17032022:1900 | Composite | 773 | 7.82 | 549.1 | 14.47 | 3.02 | 1798110 | 3.2 | 62.76 |
| Edinburgh | JR1 | 18032022:0600 | Composite | 2200 | 8.27 | 1568 | 25.03 | 4.30 | 3487300 | 4.9 | 100.00 |
| Edinburgh | JR1 | 18032022:1400 | Composite | 2053 | 7.65 | 722.1 | 20.64 | 3.43 | 1912987 | 6.5 | 33.37 |
| Edinburgh | JR1 | 18032022:2200 | Composite | 2033 | 7.93 | 754 | 22.06 | 16.90 | 1661837 | 2.5 | 30.51 |
| Edinburgh | JR1 | 19032022:0600 | Composite | 2633 | 7.31 | 791.6 | 19.08 | 18.78 | 3828570 | 5.4 | 15.62 |
| Edinburgh | JR1 | 19032022:1400 | Composite | 5160 | 8.42 | 858.3 | 33.57 | 23.15 | 3762710 | 2.7 | 41.73 |
| Edinburgh | JR1 | 19032022:2200 | Composite |  | 7.84 | 756.1 | 22.83 | 17.99 | 1151667 | 4.9 | 33.99 |
| Edinburgh | JR1 | 20032022:0600 | Composite | 9260 | 8.35 | 736.6 | 29.62 | 3.56 | 633303.3 | 2.4 | 40.11 |
| Edinburgh | JR1 | 20032022:1400 | Composite | 1167 | 7.63 | 811.9 | 22.17 | 5.71 | 778316.7 | 9.4 | 11.25 |
| Edinburgh | JR1 | 21032022:1300 | Composite |  | 7.76 | 737.7 | 24.96 | 21.01 | 552480 | 4.4 | 22.04 |
| Edinburgh | JR1 | 21032022:1900 | Composite | 1400 | 8.24 | 741.8 | 29.05 | 25.88 | 412683.3 | 3.4 | 28.15 |
| Edinburgh | JR1 | 22032022:1900 | Composite | 940 | 7.77 | 732.7 | 25.17 | 13.32 | 1374690 | 4 | 37.39 |
| Edinburgh | JR1 | 23032022:0600 | Composite | 640 | 8.60 | 934.6 | 48.88 | 22.60 | 2124997 | 3.7 | 48.49 |
| Edinburgh | JR1 | 23032022:0600 | Composite | 2680 | 8.44 | 808.6 | 36.66 | 9.23 | 1.33E+08 | 15.5 | 100.00 |
| Edinburgh | JR1 | 23032022:1300 | Composite | 467 | 8.31 | 753.7 | 30.57 | 20.77 | 69464180 | 17.3 | 100.00 |
| Edinburgh | JR1 | 23032022:1900 | Composite | 14293 | 8.45 | 887.5 | 37.00 | 19.89 | 37664243 | 14.1 | 100.00 |
| Edinburgh | JR2 | 08032022:0000 | Composite | 16973 | 7.87 | 1201 | 81.42 | 5.59 | 5737870 | 119 |  |
| Edinburgh | JR2 | 09032022:0600 | Composite | 115040 | 7.62 | 1248 | 97.71 | 5.30 | 33108760 | 121 |  |
| Edinburgh | JR2 | 10032022:0000 | Composite | 34733 | 7.35 | 1317 | 81.19 | 5.52 |  | 56.9 | 28.44 |
| Edinburgh | JR2 | 11032022:0000 | Composite | 111827 | 7.16 | 1421 | 102.61 | 5.15 |  | 64.7 | 0.66 |
| Edinburgh | JR2 | 12032022:0000 | Composite | 25080 | 7.23 | 1269 | 33.33 | 1.04 |  | 50.5 | 13.93 |
| Edinburgh | JR2 | 13032022:0000 | Composite | 3800 | 7.20 | 1475 | 102.67 | 7.13 |  | 93.5 | 1.57 |
| Edinburgh | JR2 | 14032022:0000 | Composite | 238893 | 8.14 | 1772 | 8.77 | 11.68 | 28230667 |  | 18.53 |
| Edinburgh | JR2 | 15032022:0000 | Composite | 12487 | 7.91 | 4416 | 82.51 | 5.28 | 8110067 |  | 21.54 |
| Edinburgh | JR2 | 16032022:0000 | Composite | 75433 |  |  |  |  | 1330857 |  | 33.52 |
| Edinburgh | JR2 | 18032022:0000 | Composite | 198533 | 7.67 | 1374 | 65.52 | 7.71 | 17705680 | 40.8 | 6.28 |
| Edinburgh | JR2 | 19032022:0000 | Composite | 279573 | 7.29 | 1328 | 64.21 | 30.84 | 11895237 | 92.5 | 5.09 |
| Edinburgh | JR2 | 20032022:0000 | Composite | 70307 | 6.25 | 1208 | 54.91 | 6.22 | 10120300 | 80 | 29.11 |
| Edinburgh | JR2 | 21032022:0000 | Composite | 107180 | 7.41 | 1494 | 76.65 | 8.16 | 22099917 | 52.8 | 8.56 |
| Edinburgh | JR2 | 22032022:0000 | Composite | 16360 | 7.37 | 1377 | 79.72 | 23.72 | 2203867 | 124 | 29.32 |
| Edinburgh | JR2 | 23032022:0000 | Composite | 24253 | 7.19 | 1299 | 61.56 | 32.41 | 979520 | 73.1 | 14.55 |
| Edinburgh | JR2 | 23032022:0000 | Composite | 87327 | 7.91 | 1448 | 81.76 | 9.28 | 4.45E+08 | 141 | 65.33 |
| Edinburgh | JR3 | 08032022:0000 | Composite | 16853 | 7.83 | 1611 | 138.84 | 7.32 | 16197930 | 180 |  |
| Edinburgh | JR3 | 09032022:0600 | Composite | 121853 | 7.81 | 1345 | 101.80 | 7.48 | 1.27E+08 | 135 |  |
| Edinburgh | JR3 | 10032022:0000 | Composite | 87 | 7.57 | 1010 | 23.70 | 1.32 |  | 2.4 | 48.92 |
| Edinburgh | JR3 | 14032022:0000 | Composite | 11880 | 7.96 | 1309 | 90.79 | 5.67 | 651360 |  | 9.50 |
| Edinburgh | JR3 | 15032022:0000 | Composite | 21540 | 7.50 | 1189 | 75.85 | 7.12 | 18393153 |  | 12.95 |
| Edinburgh | JR3 | 16032022:0000 | Composite | 55553 |  |  |  |  | 11153333 |  | 128.90 |
| Edinburgh | JR3 | 18032022:0000 | Composite | 716873 | 7.42 | 1259 | 59.44 | 6.59 | 16465363 | 89.5 | 39.42 |
| Edinburgh | JR3 | 19032022:0000 | Composite | 2139653 | 7.36 | 1587 | 82.81 | 26.97 | 15618200 | 54 | 10.46 |
| Edinburgh | P1 | 08032022:0000 | Composite | 13260 | 7.45 | 1342 | 52.69 | 4.76 | 18719263 | 87.9 |  |
| Edinburgh | P1 | 10032022:0000 | Composite | 31413 | 7.05 | 1400 | 53.11 | 3.20 |  | 51.2 | 9.50 |
| Edinburgh | P1 | 11032022:0000 | Composite | 36753 | 7.40 | 1626 | 49.24 | 2.35 |  | 29.5 | 0.00 |
| Edinburgh | P1 | 11032022:0600 | Composite | 96653 | 7.23 | 1528 | 57.36 | 5.68 |  | 24.1 | 0.00 |
| Edinburgh | P1 | 11032022:1800 | Composite | 17673 | 7.20 | 1459 | 72.13 | 3.84 |  | 30 | 0.00 |
| Edinburgh | P1 | 12032022:0600 | Composite | 198813 | 7.50 | 1890 | 98.91 | 5.32 |  | 39.4 | 0.00 |
| Edinburgh | P1 | 12032022:1800 | Composite | 16180 | 7.31 | 1384 | 61.39 | 2.32 |  | 40.4 | 0.00 |
| Edinburgh | P1 | 15032022:1800 | Composite | 44113 | 7.33 | 1329 | 54.34 | 2.00 | 16640727 |  | 31.20 |
| Edinburgh | P1 | 16032022:0600 | Composite | 71540 | 8.27 | 2092 | 108.27 | 5.84 | 5813910 |  | 14.06 |
| Edinburgh | P1 | 16032022:1800 | Composite | 49993 |  |  |  |  | 1965793 |  | 14.93 |
| Edinburgh | P1 | 17032022:0600 | Composite | 117400 |  |  |  |  | 1505590 |  | 9.35 |
| Edinburgh | P1 | 18032022:1700 | Composite | 70527 | 7.27 | 788.7 | 24.52 | 3.74 | 8420227 | 4.9 | 88.50 |
| Edinburgh | P1 | 21032022:1100 | Composite | 134807 | 8.39 | 2181 | 127.59 | 25.54 | 13845823 | 118 | 19.05 |
| Edinburgh | P1 | 21032022:1100 | Composite | 43940 | 7.85 | 1767 | 93.58 | 8.80 | 22996907 | 90.4 | 27.81 |
| Edinburgh | P1 | 23032022:1200 | Composite | 58107 | 7.72 | 1329 | 20.88 | 3.68 | 74825327 | 83.6 | 265.06 |
| Edinburgh | WWTP | 08032022:0000 | Composite | 238767 | 7.16 | 910.1 | 40.66 | 3.92 | 74752737 | 172 |  |
| Edinburgh | WWTP | 09032022:0600 | Composite | 140240 | 7.37 | 912.2 | 33.12 | 2.23 | 45952060 | 55.2 |  |
| Edinburgh | WWTP | 10032022:0000 | Composite | 60440 | 7.17 | 896.9 | 9.43 | 1.34 |  | 5.8 | 0.00 |
| Edinburgh | WWTP | 11032022:0000 | Composite | 92607 | 7.32 | 969.4 | 11.16 | 1.15 |  | 4.9 | 0.00 |
| Edinburgh | WWTP | 14032022:0000 | Composite | 37947 | 7.33 | 753.2 | 12.25 | 0.54 | 576733.3 |  | 31.01 |
| Edinburgh | WWTP | 15032022:0000 | Composite | 246767 | 7.31 | 755.7 | 19.03 | 2.00 | 610316.7 |  | 7.22 |
| Edinburgh | WWTP | 16032022:0000 | Composite | 370887 |  |  |  |  | 1305867 |  | 13.24 |
| Edinburgh | WWTP | 18032022:0000 | Composite | 63347 | 7.36 | 784.2 | 9.82 | 2.79 | 1541647 | 9.5 | 0.30 |
| Edinburgh | WWTP | 19032022:0000 | Composite | 1579493 | 7.29 | 909.3 | 21.13 | 23.40 | 23728120 | 15 | 23.84 |
| Edinburgh | WWTP | 22032022:0000 | Composite | 63773 | 7.44 | 767.1 | 14.98 | 3.16 | 638883.3 | 20.2 | 3.43 |
| Edinburgh | WWTP | 23032022:0000 | Composite | 16140 | 7.35 | 754.8 | 10.54 | 27.35 | 852926.7 | 4.4 | 12.07 |
| Edinburgh | WWTP | 23032022:0000 | Composite | 225680 | 7.36 | 880.6 | 14.23 | 3.20 | 1.02E+08 | 38.1 | 10.68 |
| Edinburgh | WWTP | 24032022:0000 | Composite |  | 7.40 | 838.3 | 52.35 | 4.58 | 7349397 | 18.2 | 16.41 |
| Heathrow | CTA | 08/03/2022 | Composite | 152387 |  |  |  |  | 67033110 |  | 96.07 |
| Heathrow | CTA | 09/03/2022 | Composite | 130493 | 7.98 | 2188 | 131.59 | 6.37 | 42768007 | 382 | 56.20 |
| Heathrow | CTA | 12/03/2022 | Composite | 85107 | 7.81 | 3372 | 147.97 | 5.65 |  | 82.9 | 0.00 |
| Heathrow | CTA | 13/03/2022 | Composite | 90560 | 7.81 | 3074 | 144.49 | 6.08 |  | 103 | 22.67 |
| Heathrow | CTA | 14/03/2022 | Composite | 53460 | 7.81 | 3074 | 149.64 | 6.15 |  | 98.5 | 1.37 |
| Heathrow | CTA | 15/03/2022 | Composite | 61893 | 7.19 | 4207 | 150.19 | 37.84 |  | 459 | 0.00 |
| Heathrow | CTA | 16/03/2022 | Composite | 12973 | 7.01 | 4545 | 161.26 | 75.84 | 9870557 |  | 0.00 |
| Heathrow | CTA | 17/03/2022 | Composite | 34480 | 7.35 | 2061 | 102.37 | 18.60 | 38864537 |  |  |
| Heathrow | CTA | 18/03/2022 | Composite | 66313 |  |  |  |  | 11682680 |  | 0.02 |
| Heathrow | CTA | 19/03/2022 | Composite | 251080 | 7.50 | 2276 | 75.51 | 13.53 | 24620250 | 80.4 | 0.00 |
| Heathrow | CTA | 20/03/2022 | Composite | 54353 | 7.46 | 3569 | 143.89 | 26.61 | 58290413 | 288 | 0.00 |
| Heathrow | CTA | 21/03/2022 | Composite | 87900 | 7.54 | 3487 | 99.10 | 19.78 | 43652737 | 238 | 0.00 |
| Heathrow | CTA | 22/03/2022 | Composite | 69920 | 7.70 | 2618 | 113.73 | 12.18 | 20838623 | 82 | 0.69 |
| Heathrow | CTA | 23/03/2022 | Composite | 39427 | 7.58 | 2950 | 129.52 | 41.64 | 17802457 | 263 | 32.42 |
| Heathrow | CTA | 24/03/2022 | Composite | 41147 | 7.95 | 3780 | 130.15 | 41.57 | 50658110 | 150 | 6.62 |
| Heathrow | VT | 16032022:0955 | Grab | 740 | 8.81 | 9935 | 143.89 | 50.52 | nd | NA |  |
| Heathrow | VT | 16032022:1025 | Grab | nd | 9.31 | 13.81 | 118.02 | 122.64 | nd | NA |  |
| Heathrow | VT | 16032022:1230 | Grab | 2378413 | 8.85 | 1952 | 54.24 | 117.53 | nd | NA |  |
| Heathrow | VT | 16032022:1300 | Grab | 7464367 | 9.00 | 45.24 | 106.92 | 69.83 | nd | NA |  |
| Heathrow | VT | 16032022:0810 | Grab | 69953 | 7.89 | 18.65 | 69.51 | 135.68 | 7292740 | NA |  |
| Heathrow | VT | 16032022:0840 | Grab | 1312407 | 8.62 | 13.57 | 144.57 | 99.07 | nd | NA |  |
| Heathrow | VT | 16032022:0910 | Grab | nd | 9.11 | 23.29 | 42.37 | 168.55 | 66942797 | NA |  |
| Heathrow | VT | 16032022:0940 | Grab | 733 | 9.34 | 18.4 | 75.34 | 184.45 | nd | NA |  |
| Heathrow | VT | 16032022:1330 | Grab | 1625460 | 8.72 | 14.07 | 85.47 | 57.88 | 75997880 | NA |  |
| Heathrow | VT | 16032022:1400 | Grab | 153567 | 8.72 | 19.18 | 47.85 | 91.00 | 87873650 | NA |  |
| Bristol | MH2 | 21032022:1000 | Composite | 19833 | 8.75 | 2369 | 131.07 | 5.44 | 8445270 | 6.9 | 100.00 |
| Bristol | MH2 | 21032022:1330 | Composite | 19320 | 8.80 | 2141 | 112.69 | 6.84 | 20601633 | 43 | 66.43 |
| Bristol | MH2 | 21032022:1700 | Composite | 25513 | 8.87 | 2880 | 156.03 | 7.54 | 33916130 | 44 | 80.28 |
| Bristol | MH2 | 21032022:2030 | Composite | 9573 | 8.94 | 2264 | 139.24 | 7.61 | 25163317 | 22.2 | 100.00 |
| Bristol | MH2 | 22032022:0530 | Composite | 43740 | 8.87 | 1888 | 98.31 | 19.32 | 25416817 | 27.8 | 100.00 |
| Bristol | MH2 | 24032022:0530 | Composite | 37760 | 8.56 | 1343 | 52.23 | 27.43 | 6092713 | 82 | 46.89 |
| Bristol | MH2 | 24032022:1000 | Composite | 163480 | 8.29 | 1690 | 95.41 | 12.72 | 211736.7 | 444 | 0.00 |
| Bristol | MH2 | 25032022:0530 | Composite | 304947 | 8.84 | 3317 | 124.81 | 4.47 | 16091543 | 1501 | 13.09 |
| Bristol | MH2 | 25032022:1000 | Composite | 60473 | 8.75 | 2025 | 125.32 | 5.21 | 1919847 | 162 | 34.54 |
| Bristol | MH2 | 26032022:0530 | Composite | 857073 | 8.35 | 1359 | 83.71 | 10.42 | 1.64E+08 | 68.2 | 78.35 |
| Bristol | MH2 | 26032022:1000 | Composite | 266173 | 8.79 | 2416 | 145.33 | 6.10 | 2246113 | 27.2 | 41.23 |
| Bristol | MH2 | 26032022:1330 | Composite | 449267 | 8.77 | 1789 | 98.53 | 4.74 | 2851840 | 21.1 | 39.47 |
| Bristol | MH2 | 26032022:1700 | Composite | 699627 | 8.97 | 8502 | 142.65 | 4.86 | 70912837 | 388 | 120.37 |
| Bristol | MH2 | 27032022:0530 | Composite | 127453 | 8.61 | 1655 | 114.54 | 6.14 | 32706533 | 677 | 0.00 |
| Bristol | MH2 | 27032022:1000 | Composite | 133167 | 9.05 | 2693 | 159.72 | 5.12 | 10136000 | 198 | 3.12 |
| Bristol | MH2 | 27032022:1330 | Composite | 73047 | 8.94 | 2363 | 127.39 | 4.44 | 3733947 | 2000 | 33.01 |
| Bristol | MH2 | 27032022:1700 | Composite | 61060 | 8.22 | 2228 | 118.00 | 5.60 | 1723820 | 517 | 0.00 |
| Bristol | MH2 | 27032022:2030 | Composite | 146600 | 9.17 | 2698 | 135.63 | 7.42 | 29471853 | 50.2 | 33.59 |
| Bristol | MH2 |  | Composite | 345093 |  |  |  |  | 28755380 |  | 78.74 |
| Bristol | MH2 | 28032022:1000 | Composite | 148187 | 8.73 | 1718 | 112.44 | 4.59 | 6899603 | 453 | 46.07 |
| Bristol | MH2 | 28032022:1330 | Composite | 184627 | 8.71 | 2045 | 106.87 | 5.86 | 444926.7 | 271 | 0.00 |
| Bristol | MH2 |  | Composite | 11953 | 8.63 | 3427 | 158.38 | 15.00 | 1760160 | 560 | 0.00 |
| Bristol | MH2 | 28032022:1700 | Composite | 65273 | 9.06 | 2143 | 112.78 | 6.52 | 984516.7 | 218 | 25.30 |
| Bristol | MH2 | 28032022:2030 | Composite | 130547 | 9.06 | 2504 | 130.27 | 7.07 | 64842310 | 646 | 0.00 |
| Bristol | MH3 | 21032022:1000 | Composite | 102127 | 7.83 | 1657 | 64.39 | 32.82 | 1.46E+08 | 72.7 | 48.70 |
| Bristol | MH3 | 21032022:1330 | Composite | 149973 | 7.71 | 1682 | 64.03 | 25.85 | 2.85E+08 | 108 | 46.26 |
| Bristol | MH3 | 22032022:0530 | Composite | 66133 | 7.99 | 1400 | 50.91 | 27.27 | 4.87E+08 | 187 | 100.00 |
| Bristol | MH3 | 24032022:0530 | Composite | 500880 | 7.59 | 2010 | 92.95 | 9.98 | 84618640 | 52.6 | 2.25 |
| Bristol | MH3 | 24032022:1330 | Composite | 4170447 | 8.83 | 4573 | 152.23 | 14.84 | 81595547 | 1877 | 31.98 |
| Bristol | MH3 | 24032022:1700 | Composite | 226587 | 7.62 | 1658 | 80.95 | 7.72 | 1131790 | 66.9 | 14.31 |
| Bristol | MH3 | 24032022:2030 | Composite | 62267 | 8.62 | 1802 | 145.11 | 5.83 | 2190100 | 120 | 22.07 |
| Bristol | MH3 | 25032022:0530 | Composite | 460693 | 7.66 | 2375 | 101.26 | 7.62 | 57005713 | 194 | 34.34 |
| Bristol | MH3 | 25032022:1000 | Composite | 213453 | 7.83 | 2040 | 109.75 | 9.09 | 11082717 | 61.7 | 3.28 |
| Bristol | MH3 | 25032022:1330 | Composite | 291400 | 8.07 | 2117 | 132.75 | 10.09 | 8460563 | 95.5 | 3.21 |
| Bristol | MH3 | 25032022:1700 | Composite | 251920 | 8.37 | 1844 | 93.33 | 6.81 | 2407287 | 31.8 | 16.37 |
| Bristol | MH3 | 26032022:0001 | Composite | 122433 | 7.81 | 1818 | 87.43 | 8.46 | 2534357 | 52.9 | 4.72 |
| Bristol | MH3 | 26032022:0530 | Composite | 194453 | 7.74 | 1629 | 98.19 | 11.27 | 14887597 | 1705 | 0.00 |
| Bristol | MH3 | 26032022:1000 | Composite | 446440 | 7.66 | 1970 | 122.52 | 13.01 | 5299807 | 293 | 0.42 |
| Bristol | MH3 | 26032022:1330 | Composite | 1751233 | 7.98 | 2180 | 141.52 | 8.05 | 6993817 | 45.9 | 0.10 |
| Bristol | MH3 | 26032022:1700 | Composite | 1246140 | 7.89 | 1927 | 105.13 | 12.28 | 86256740 | 85.1 | 5.25 |
| Bristol | MH3 | 26032022:2030 | Composite | 164373 | 8.45 | 1717 | 112.05 | 6.48 | 4090860 | 28.1 | 39.73 |
| Bristol | MH3 | 27032022:0530 | Composite | 113507 | 9.02 | 2362 | 66.55 | 7.92 | 3672967 | 124 | 1.79 |
| Bristol | MH3 | 28032022:0530 | Composite | 835300 | 8.73 | 1654 | 111.12 | 4.36 | 16365573 | 588 | 96.67 |
| Bristol | MH3 | 27032022:1000 | Composite | 203407 | 8.39 | 2473 | 103.56 | 6.99 | 4529390 | 695 | 20.09 |
| Bristol | MH3 | 27032022:1330 | Composite | 106380 | 7.75 | 1905 | 104.70 | 12.43 | 10664900 | 489 | 1.26 |
| Bristol | MH3 | 27032022:1700 | Composite | 42540 | 8.38 | 2416 | 142.08 | 9.92 | 3024537 | 70.3 | 0.00 |
| Bristol | MH3 | 27032022:2030 | Composite | 301960 |  |  |  |  | 25881607 |  | 9.08 |
| Bristol | MH3 | 29032022:0530 | Composite | 170600 | 8.53 | 1783 | 106.42 | 9.44 | 9765797 | 341 | 0.00 |
| Bristol | MH3 | 28032022:1000 | Composite | 198987 | 7.40 | 1948 | 116.43 | 14.94 | 7106423 | 1110 | 0.00 |
| Bristol | MH3 | 28032022:1330 | Composite | 190947 | 8.13 | 2145 | 143.14 | 8.08 | 14421480 | 354 | 0.00 |
| Bristol | MH3 | 28032022:1700 | Composite | 56927 | 8.05 | 1935 | 136.89 | 10.17 | 4997450 | 822 | 0.00 |
| Bristol | MH3 | 29032022:0530 | Composite | 623027 | 7.60 | 1676 | 89.16 | 11.48 | 99206427 | 3749 | 13.28 |
| Bristol | MH1 | 25032022:1500 | Composite | 3314440 | 9.13 | 21390 | 53.95 | 161.92 | 22758280 | 1820 | 0.00 |
| Bristol | MH1 | 26032022:0300 | Composite | 53453 | 7.87 | 1186 | 45.31 | 6.48 | 2187670 | 29.4 | 10.12 |
| Bristol | MH1 | 26032022:0430 | Composite | 17007693 | 9.26 | 14240 | 130.84 | 100.31 | 2.69E+08 | 2389 | 0.00 |
| Bristol | MH1 | 25032022:1735 | Composite | 5287587 | 9.00 | 18130 | 126.96 | 118.37 |  | 2614 | 0.00 |
| Bristol | MH1 | 27032022:0000 | Composite | 3207 | 9.38 | 17870 | 49.78 | 160.04 |  | 757 | 0.00 |
| Bristol | MH1 | 27032022:1827 | Composite | 37353 | 9.29 | 14600 | 104.32 | 88.68 | 11115160 | 822 | 0.00 |
| Bristol | MH1 | 28032022:0900 | Composite | 2456100 | 7.53 | 1609 | 74.68 | 19.74 | 66962247 | 1441 | 79.50 |
